# Linking Bacterial Communities Associated with the Environment and the Ecosystem Engineer *Orchestia gammarellus* at Contrasting Salt Marsh Elevations

**DOI:** 10.1007/s00248-020-01656-w

**Published:** 2021-01-09

**Authors:** Edisa García Hernández, Matty P. Berg, A. Raoul Van Oosten, Christian Smit, Joana Falcão Salles

**Affiliations:** 1 Microbial Community Ecology Group, Groningen Institute for Evolutionary Life Sciences, University of Groningen, Groningen, Netherlands; 2 Department of Ecological Sciences, Section Animal Ecology, Vrije Universiteit Amsterdam, Amsterdam, Netherlands; 3 Conservation Ecology Group, Groningen Institute for Evolutionary Life Sciences, University of Groningen, Groningen, Netherlands

**Keywords:** *Orchestia gammarellus*, Talitridae, Salt marsh elevation, Soil fauna, Ecosystem engineer, Gut microbiome

## Abstract

The digestive tract of animals harbors microbiota important for the host’s fitness and performance. The interaction between digestive tract bacteria and soil animal hosts is still poorly explored despite the importance of soil fauna for ecosystem processes. In this study, we investigated the interactions between the bacterial communities from the digestive tract of the litter-feeding, semi-terrestrial crustacean *Orchestia gammarellus* and those obtained from the environment; these organisms thrive in, i.e., soil and plant litter from salt marshes. We hypothesized that elevation is an important driver of soil and litter bacterial communities, which indirectly (via ingested soil and litter bacteria) influences the bacterial communities in the digestive tract of *O. gammarellus*. Indeed, our results revealed that elevation modulated soil and litter bacterial community composition along with soil organic matter content and the C:N ratio. Soil and plant litter differed in alpha diversity indexes (richness and diversity), and in the case of plant litter, both indexes increased with elevation. In contrast, elevation did not affect the composition of bacterial communities associated with *O. gammarellus*’ digestive tract, suggesting selection by the host, despite the fact that a large component of the bacterial community was also detected in external sources. Importantly, *Ca. *Bacilloplasma and *Vibrio* were highly prevalent and abundant in the host. The taxonomic comparison of *Ca. *Bacilloplasma amplicon sequence variants across the host at different elevations suggested a phylogenetic divergence due to host habitat (i.e., marine or semi-terrestrial), thus supporting their potential functional role in the animal physiology. Our study sheds light on the influence of the environment on soil animal–bacteria interactions and provides insights into the resilience of the *O. gammarellus*–associated bacteria to increased flooding frequency.

**Supplementary Information:**

The online version contains supplementary material available at 10.1007/s00248-020-01656-w.

## Introduction

Most multicellular organisms have a tight symbiotic relationship with microbes, for instance, in their digestive tract. Digestive tract microbiomes provide unique metabolic functions to the host, in addition to the already existing host’s metabolism. The digestive tract microbiome has also been linked to other host traits, such as mating preference, longevity, and reproduction [1, 2]. Therefore, the outcome of the commensal and mutualistic interactions between the host and its digestive tract microbiome has an effect on the host performance and fitness [3], which may have an effect on ecosystem processes. The fitness of the holobiont, which consists of the host plus its microbiome [4], is particularly crucial for ecosystems when the holobiont is able to modulate the availability of resources to other species, i.e., as in the case of soil ecosystem engineers [5], which promote soil aeration and nutrient mineralization through their burrowing activity (bioturbation) [6]. Despite their pivotal importance for soil structure and fertility, little is known about the digestive tract microbiome of soil ecosystem engineers and how the internal microbial composition influences the performance of these key soil animals [7–9].

The composition of the microbiome from the digestive tract of litter-feeding ecosystem engineers, such as earthworms, isopods, and millipedes, is shaped by internal and external factors. Internal factors relate to the host genotype, which comprises the genes that allow microbial colonization of the digestive organs [10] and determine the host physiological conditions that permit the establishment of microbes, as well as the quality of resources that support microbial growth, via preferential feeding [11]. Moreover, developmental stage, hormones, and immune response are modulators of gut microenvironment in invertebrates [12, 13]. For instance, local conditions differ along the digestive tract sections, such as pH and oxygen concentration in the foregut, midgut, and hindgut, which modulate enzyme activity and microbial composition [13]. These internal factors together act as a selective force that filters the microbes from the exterior into the digestive tract, thus restraining microbial composition and explaining the specific microbiomes of terrestrial amphipods [8, 14] and isopod species [15]. External factors are associated with the environmental pool of microorganisms; the macrodetritivore hosts can interact with, i.e., the microbial communities in bulk soil and in plant litter. For instance, soil pH, temperature, and C/N ratio explain microbial diversity in a great variety of soils [16, 17]. Similarly, plant litter chemical traits, such as total carbon, total nitrogen, and lignin content, determine the microbial diversity and composition on its surface [18].When ingested, the microbes on the litter can be assimilated as food or they might deliver extracellular enzymes in the digestive tract that facilitate the digestion of plant litter and impact in the host’s growth [11, 19, 20]. However, we still lack an understanding on how internal and external factors interact and whether they contribute, alone or in concert, to the composition of digestive microbes in macrodetritivore tracts.

The terrestrial amphipod *Orchestia gammarellus* (Pallas 1766) (Crustacea, Talitridae) plays a key role in salt marshes, but its microbiome is poorly understood [14]. This ecosystem engineer significantly affects soil pore space formation, the fragmentation and decomposition of litter, and nutrient cycling and is the most abundant macrodetritivore of many salt marshes in Western Europe [21]. Its distribution is strongly linked to abiotic conditions, particularly soil moisture, salinity, and temperature [21, 22], being mostly found at low to intermediate salt marsh elevations where the environmental conditions are suitable. At low elevation, daily salt water inundations restrict the occurrence of this species, while at high elevation, summer drought limits their distribution on the salt marsh. Similarly, salt marsh soil microbes and plants are distributed according to their tolerances to tidal inundation differences [23, 24]. Thus, elevation is also determining the composition of the plant litter microbiome that *O. gammarellus* ingests, shaping indirectly the digestive tract microbiome [14]. Taken together, we expect local environments to have an impact on the host–microbiome relationship, either directly—through *O. gammarellus* physiology and response to stressful environmental conditions, which might influence microbiome colonization—and indirectly, via environmental microbes.

Here, we study the indirect effect of elevation on the bacterial communities of the digestive tract of *O. gammarellus* (ODT, hereafter) via interactions with bacteria in bulk soil and plant litter. In salt marsh ecosystems, elevation determines inundation frequency [25] and, hence, soil physicochemical properties and plant litter quality due to shifts in vegetation composition. We thus expect that these elevation-induced changes will have an effect on the soil- and litter-inhabiting microbes *O. gammarellus* feeds on or is in contact with. Our specific objectives were (1) to assess the differences in bacterial communities across bulk soil, plant litter, and ODT, (2) to assess the effect of elevation on these bacterial communities and their interactions, (3) to determine if a stable core ODT bacterial composition exists, and (4) to determine the phylogenetic relationship between the dominant species in the digestive tract, *Ca. *Bacilloplasma, and other *Bacilloplasma* -like species from other hosts. Together, these objectives allowed us to quantify the influence of external sources on the internal microbiome of this important soil ecosystem engineer.

## Material and Methods

### Study Site and Plot Description

This study was performed on the salt marsh of the barrier island of Schiermonnikoog, the Netherlands (53° 29′ N, 6° 10′ E). The sampling was done on October 4–5, 2017. During that month, the average temperature was 14 °C and the mean precipitation in September was 41.5 mm (worldweatheronline.com). On this island, a well-documented salt marsh chronosequence is present [26].

The elevation of each site was measured in nine randomly selected points using a real-time kinematic differential Global Positioning System (RTK-dGPS, Leica Viva GS12 GNSS receiver and CS15 controller), with a vertical accuracy of less than 2 mm. Inundation frequency and duration for each site were calculated using a regression model [27], based on site elevation and the actual sea water level. Three sites (A, B, or C) were selected at high elevation (HE) (> 1.450 mAOD, Amsterdam Ordnance Data) and low elevation (LE) (< 1.270 mAOD) (Supplement S1, Table 1). The estimated flooding frequency at LE sites was approximately two times higher than that at HE sites (Table 1). At each site, triplicate 3 × 3 m^2^ plots separated by a distance more than 2 m were laid out. The vegetation of the salt marsh varied with elevation (Table 1), and the vegetation successional stage of each site was inferred according to Schrama et al. [28]. It is important to note that the term “elevation,” used throughout this manuscript, implies differences in the frequency and duration of flooding by seawater and hence variations in physicochemical composition of soil and relative abundance of plant species.

**Table 1:** Sample sites and some basic characteristics at the salt marsh of Schiermonnikoog, the Netherlands. Elevation is given as mean Amsterdam Ordnance Data (mAOD) units. Chronosequence age is expressed in years after establishment. Flooding frequency is expressed as the annual proportion of inundated time in hours. At each site, the dominant vegetation was recorded

Site	Latitude	Longitude	Elevation (mAOD)	Chronosequence age (years)	Flooding frequency	Dominant vegetation
High A	53.48979	6.22712	1.486	78	0.046	*Elytrigia atherica*, *Atriplex prostrate*
High B	53.49203	6.26539	1.461	53	0.050	*E. atherica, Festuca rubra*, *Artemisia maritima*
High C	53.49659	6.2769	1.498	53	0.044	*Limonium vulgare*, *F. rubra*
Low A	53.47791	6.23982	1.216	31	0.138	*E. atherica*, *A. maritima*, *Atriplex portulacoides*, *Suaeda marítima*
Low B	53.48484	6.26841	1.263	31	0.112	*E. atherica*, *A. maritima*, *L. vulgare*
Low C	53.48913	6.2757	1.254	24	0.116	*E. atherica*, *A. maritima*, *L. vulgare*

### Sampling and Measurements of Physicochemical Parameters of Soil and Plant Litter

Sixteen cores of bulk soil (Ø 5 cm: 3 cm depth) sampled at random points within each plot were pooled in a sterile plastic bag, which was sealed, kept cool, and transported to the laboratory on the same day. In the laboratory, the soil was sieved (4 mm mesh size) to represent a composite sample for each plot (3 sites × 2 elevations × 3 plots = 18). Ten grams of soil was placed in sterile tubes and frozen at − 20 °C for DNA extraction. Approximately 200 g soil per sample was kept at 4 °C for physicochemical measurements. Differences in soil physicochemical parameters, i.e., soil moisture content, soil organic matter content (SOM), and the content of sodium (Na), total carbon (TC), total nitrogen (TN), and N–NO_3_^−^, N–NO_2_^−^, and N–NH_4_^+^, were quantified using the methodology described in Appendix A1.

We collected all *O. gammarellus* individuals and all plant litter laying on the soil from the interior of a plastic core (Ø = 17 cm) by hand. Around 2.5 g of litter was put in a paper bag for C/N ratio measurement and *O. gammarellus* in 70% ethanol. Moreover, around 10 g of litter from within each plot (outside the core) was placed in sterile tubes for microbial extraction, which were kept at 4 °C and processed within 24 h.

### Microbial Extraction of Plant Litter and Extraction of *O. gammarellus* Digestive Tract Samples

To extract plant litter microbes, 5 g of litter was cut to 0.5 cm fragments with sterilized scissors and placed in flasks with 45 ml of sterilized 0.1% Na_4_P_2_O_7_ containing ~ 20 sterile 3-mm glass beads. The flasks were shaken (200 rpm) at room temperature (25 °C) for 1 h. The content was then transferred to 50-ml sterile tubes and thoroughly mixed using a vortex for 5 min at full speed. Plant material was removed, and the suspension was transferred to a new sterile tube and centrifuged at 3200 × *g* for 15 min, after which the pellet was stored at − 20 °C.

Ten *O. gammarellus* adults (stages with 13–16 podomeres) from each plot were randomly selected for digestive tract (ODT) extraction, except for one plot of LC, HB, and HC where nine, six, and seven individuals were selected, respectively. Each animal was washed in 10 ml sterilized water, then two times in 10 ml 70% ethanol, and two times in sterilized water. After that, the ODT (from the stomach to the anus) was extracted in aseptic conditions under a stereoscope with 16x time magnification inside a flow cabinet. All the equipment used was sterilized using a flame and then washed in DNA AWAY® (Molecular BioProducts, San Diego, CA) after the dissection of each specimen. The ODT dissected were pooled per plot (3 plots × 3 sites × 2 elevations = 18 pools) in a 1.5-ml tube with 500 μl of 0.85% NaCl solution and frozen at − 20 °C until DNA extraction.

### DNA Isolation and Partial 16S rRNA Gene Sequencing

We extracted DNA from bulk soil, plant litter, and ODT samples from 0.25 g of soil, 0.25 g pellet, or 0.5 ml of digestive tract solution, respectively, using the DNeasy Power Soil (Qiagen) extraction kit. The manufacturer’s instructions were followed, except for the addition of 0.2 g of 0.1-mm sterile glass beads to enhance cell lysis. The amount of extracted DNA was quantified using a NanoDrop spectrophotometer (Thermo Scientific, USA). We partially amplify the 16S rRNA gene using the 515F–926R primer set, followed by pooling of the amplicons in equimolar concentration and sequencing on an Illumina MiSeq sequencer using a 2 × 300-bp read configuration. The details on sample preparation for sequencing can be found in Appendix A2.

### Sequence Analyses

To join the paired-end sequences, we used the Quantitative Insights into Microbial Ecology (QIIME) version 1.91 (function join_paired_ends.py) [29]. Demultiplexing and removing of primers were performed using the sequencing cutadapt toolkit [30]. Demultiplexed sequences were then imported into QIIME2 version 2018.2 and were quality filtered using the deblur algorithm [31] following the default parameters [32] except that the amplicons were trimmed to 380 bp length. The reads showed high-quality score (*q* > 35). Taxonomic identity to the amplicon sequence variants (further on ASVs) was assigned using the reference database SILVA (version 132-2018) trained for the 515F/926R region with a default similarity threshold of 0.7. Resultant outputs, i.e., feature table, taxonomy table, and phylogenetic tree, were then imported into R (R 3.6.1, http://www.r-project.org).

Further sequence analyses were done using the Phyloseq package [33]. Singletons, ASVs with non-assigned phylum, and ASVs identified as mitochondria, chloroplast, and archaea were discarded (Apx. A3). The resulting ASV table was then used for the subsequent analyses. A rarefaction to an even sampling depth of 3000 reads was performed to all the samples (Supplement S2). One plant litter sample from the site HA and two digestive tract samples from the site LA were excluded from the analyses because they had low read numbers. The selected set of rarefied sequences was then used to calculate α-diversity metrics, namely, ASV richness (observed ASVs), Shannon’s diversity index, and Faith’s phylogenetic diversity. To assess if α-diversity differed between bulk soil, plant litter, and ODT, we used ANOVA, followed by pairwise comparisons using least squared means with Tukey’s multiple comparison test implemented in emmeans [34] (ODT, *N* = 6; PL, *N* = 6; and soil, *N* = 6). Differences in α-diversity metrics due to elevation in each bacterial source were assessed using linear mixed models, with the site as a random factor and pairwise comparison using least squared means with false discovery rate (FDR) *p* adjustment method.

To assess the variation in ASV composition between the types of source (soil, plant litter, and ODT), the ASV tables were normalized at relative abundances prior to calculating the Bray–Curtis compositional dissimilarity between samples, and weighted and unweighted UniFrac distance matrices were constructed using the package vegan [35, 36] and visualized in PCoA plots. Significant differences in microbial structure were tested using PERMANOVA with the function adonis from the vegan package. All significant results were tested for data dispersion using the function betadisper. Moreover, we tested the turnover and nestedness components of β-diversity (ASV presence/absence) between the types of sources by estimating Sørensen-based multiple-site dissimilarity (*β*_SOR_) [37] implemented in the R package betapart [38]. The turnover component (replacement) was measured as Simpson pairwise dissimilarity (*β*_-SIM_) and the nestedness measured as the nestedness fraction of Sørensen pairwise dissimilarity (*β*-_SNE_) [38]. A similar approach was used for testing phylogenetic turnover and nestedness components of phylogenetic β-diversity based on Faith’s phylogenetic diversity. In this case, turnover and nestedness dissimilarity matrices were measured as their respective fractions of Jaccard pairwise phylogenetic dissimilarity (UniFrac index) [36].

To assess the effect of inundation on the bacterial communities in ODT, plant litter, and soil, we first compared the bacterial phylogenetic structure using the phylogenetic isometric log-ratio transform (PhIlR), which takes ratios on a bifurcating phylogenetic tree [39]. Taxa that were not seen in at least 10% of the samples and with not more than two counts were discarded, and then, the filtered ASV datasets were PhIlR transformed using the philr package. Euclidean distance matrices were calculated on the PhILR-transformed datasets and visualized using PCoA plots using the tools of the phyloseq package. The elevation effect on sample dissimilarity distances was tested using PERMANOVA with the *adonis* function [40]. To test which soil physicochemical parameters explained observed differences in the soil bacterial community composition, we compared the Bray–Curtis distance matrix (bacteria) with the Euclidean distance matrix (soil parameters). The best subset of soil parameters that explained observed variance in bacterial community data was obtained using the Spearman correlation method with the function *bioenv* implemented in the vegan package. The Bray–Curtis dissimilarity matrix was used as input for a NMDS biplot using the metaMDS function, and the significant soil parameters were fitted using the *envfit* function in the vegan package.

The description of the core digestive tract bacterial community was based on ASVs present in 90% of the ODT samples and which accounted for at least 0.0001% of the total ASV relative abundance in these samples. We further compared the relative abundance of the core community in ODT samples between elevations using the Wilcoxon rank-sum test. Moreover, we visualized the relative abundance at the genus level of the core ODT in external sources in bar plots. To disentangle the proportion of the ASVs exclusively found in ODT and ASVs shared with environmental sources and elevations, we performed a Venn diagram in which the percentages were calculated using the total ODT ASV dataset. Taxonomic abundance of exclusively and shared ASVs was filtered and visualized in bar plots comparing elevations. All amplicon sequences are publicly accessible on the NCBI database under the BioProject ID PRJNA602740.

### Phylogenetic Relationship of *Ca. *Bacilloplasma Found in ODT with Other Hosts

The sequences that were identified as Ca. Bacilloplasma were aligned to the most similar sequences downloaded from the NCBI repository (ncbi.nlm.nih.gov) using ClustalW. A phylogenetic tree was reconstructed using the maximum likelihood method and Tamura–Nei model. Tree reliability was estimated with a bootstrap method using 1000 iterations. For the tree reconstruction and visualization, we used MEGA X [41].

## Results

### Bacterial Diversity and Composition of Bulk Soil, Plant Litter, and *O. gammarellus* Digestive Tract

**Fig. 1:**
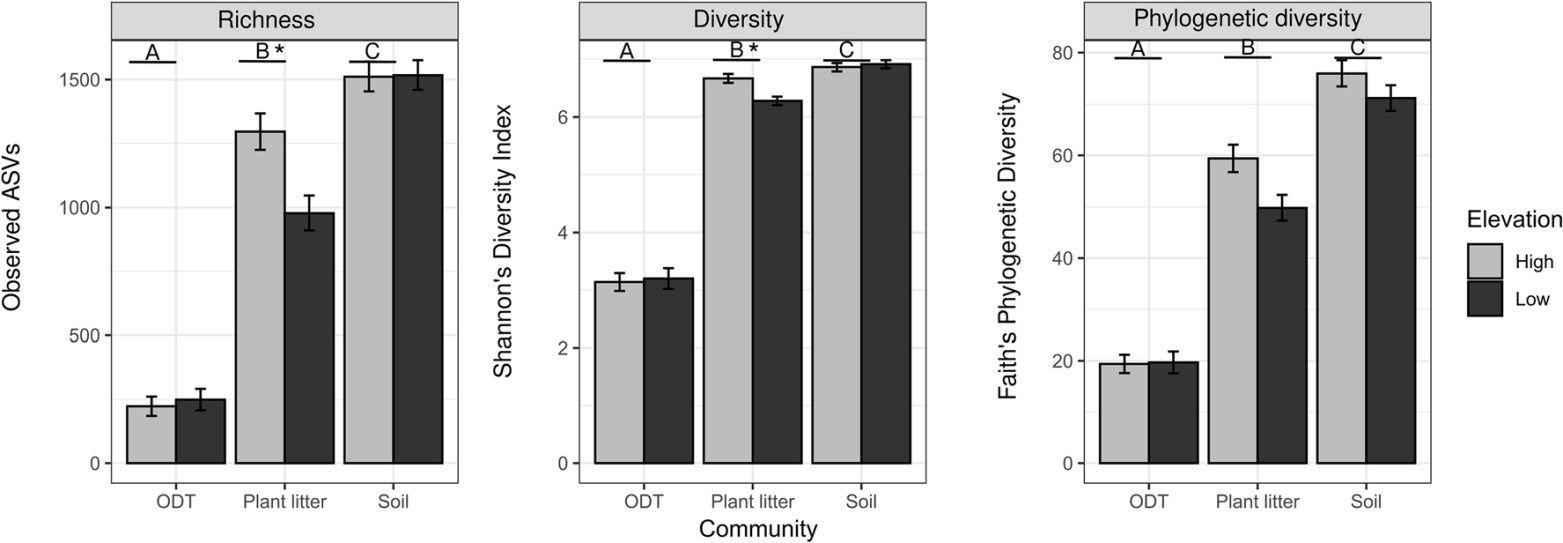
Alpha diversity metrics of soil, plant litter, and *Orchestia gammarellus* digestive tract (ODT) bacterial communities. Least squared means ± SE from linear model analysis of the amplicon sequence variant (ASV) richness, diversity, and phylogenetic diversity at high and low elevations. Different letters depict significant differences (Tukey’s multiple comparison test adj *p* < 0.05) between community sources. Asterisk indicates significant difference between elevations within a source (FDR,adj *p* < 0.05)

A total of 1,048,661 reads were obtained after the removal of non-target sequences (2.5% of the sequences were removed), leading to a final dataset that included 34,257 unique ASVs. Soil and plant litter (PL) harbored 84.6% and 79.3% more ASVs than ODT, respectively, and ASV richness was significantly different between sources (ANOVA, *F*_(df = 2)_ = 135.8, *p* < 0.001; Fig. 1; for pairwise comparisons, see Supplement S3). Shannon and phylogenetic diversities followed the same pattern with the highest values in soil, intermediate in plant litter, and lowest in ODT and were significantly different between the three of them (Shannon’s diversity index, ANOVA, *F*_(df = 2)_ = 585.16, *p* < 0.001); PD, ANOVA, *F*_(df = 2)_ = 195.7, *p* < 0.001; for pairwise comparisons, see Supplement S3).

**Fig. 2:**
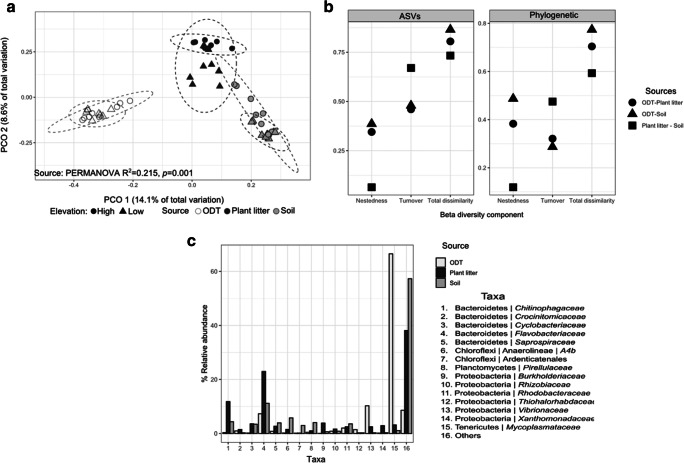
Differences in bacterial community structure and composition between *O. gammarellus* digestive tract (ODT), plant litter, and soil. **a** Principal coordinate analysis (PCoA) based on unweighted UniFrac distances. Ellipses indicate similarity at 95% confidence intervals of elevations. **b** Total β-diversity (Sørensen index) and its nestedness and turnover components comparing community sources. The difference between ASV diversity and phylogenetic diversity is that the former is based on the ASV table and the latter on the phylogenetic tree. **c** Relative abundance profile of the 15 bacterial taxa with the highest values in the Dirichlet multinomial mixtures model, which are driving differences between community sources

PCoA based on unweighted UniFrac distances revealed segregation in bacterial composition from the different source types. PCO1 explained 14.1% of the variances in phylogenetic structure (Fig. 2a) and clearly separated the three community sources (PERMANOVA, *R*^2^ = 0.215, *p* = 0.001). Moreover, the interaction between source and elevation (PERMANOVA, *R*^2^ = 0.054, *p* < 0.001) and elevation alone (PERMANOVA, *R*^2^ = 0.040, *p* < 0.001) also explained some variance. Sources showed a homogeneous variance (PERMANOVA, *p* = 0.603), suggesting that differences between sources rather than dispersion of the data explained the variance. A similar pattern was observed for ASV relative abundances using Bray–Curtis and lineage abundance using weighted UniFrac distances (Supplements S4 and S5). Compositional differences between sources were attributed to both turnover (species replacement) and nestedness (species loss) components of β-diversity (Fig. 2b). Regarding relative abundances in ASVs (Bray–Curtis), differences between sources were mostly due to turnover and to a lesser extent nestedness. Thus, ODT harbors exclusive ASVs but also is a subset of the litter and soil communities. However, the establishment of phylogenetic relationships between ASVs revealed that the between-source β-diversity involving ODT samples was explained mainly by nestedness rather than turnover, indicating that many of the ASVs found in ODT belonged to bacterial lineages also found in environmental samples. Therefore, dissimilarity was explained by a loss of bacterial lineages that are present in the environment but restricted in ODT.

We conducted cluster analyses of the samples to identify “envirotype” assemblages. Three clusters were identified by the DMM model which coincided with the three types of source (Supplement S6). The taxa with the highest values of the model indicate their relevance in driving differences between the envirotypes. The ODT envirotype was predominantly defined by *Mycoplasmataceae* and *Vibrionaceae*, the plant litter group by *Flavobacteriaceae* and *Chitinophagaceae*, and the soil envirotype by *Flavobacteriaceae* and *Anaerolineaceae* (Fig. 2c, Supplement S6).

### Effect of Elevation on Salt Marsh Bacterial Communities

Elevation had an effect on ASV richness and Shannon’s diversity only in plant litter, with higher ASV richness and diversity at high elevation (HE, hereafter) (richness: lsmeans, *t*_(df = 3.8)_ = 3.23, *p* = 0.03; Shannon’s index lsmeans, *t*_(df = 3.96)_ = 3.64, *p* = 0.02) (Fig. 1, Supplement S7).

**Fig. 3:**
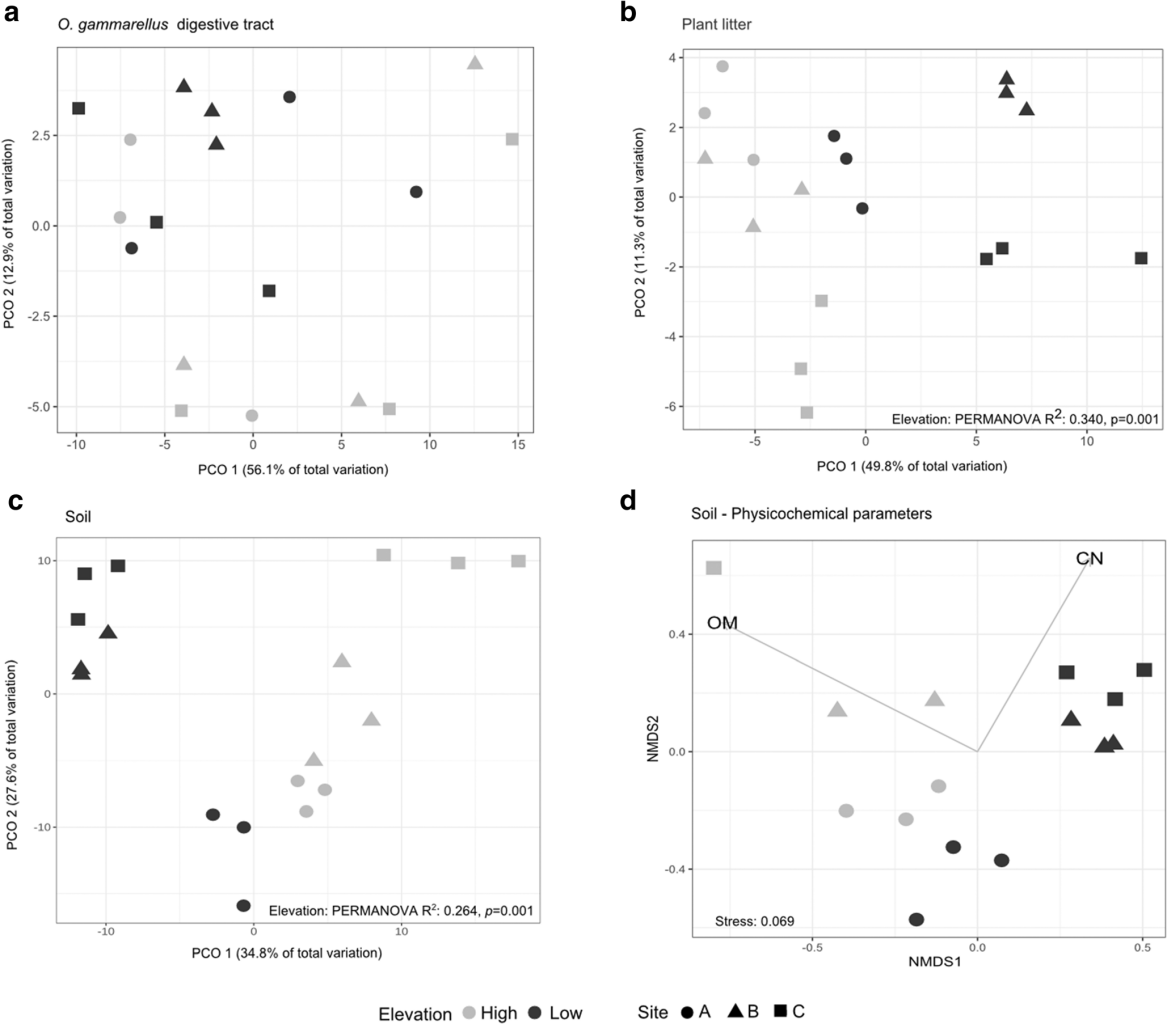
Effect of elevation on bacterial phylogenetic composition in each source. PCoA displaying sample Euclidean distances computed in the PhILR coordinate system in the *O. gammarellus* digestive tract (**a**), plant litter (**b**), and soil samples (**c**). NMDS plot showing the correlation between bacterial ASVs of soil and the best soil physicochemical parameters that describe their Bray–Curtis dissimilarity distance. The vectors represent the mean direction and strength of the Spearman correlation of the parameters measured (**d**)

The similarity in bacterial phylogenetic composition between elevations depended on the type of source. Elevation did not affect ODT bacterial composition (PERMANOVA nperm = 999, *R*^2^ = 0.092, *p* = 0.152) (Fig. 3a) but did affect the composition of plant litter and soil bacterial communities (Fig. 3b, c; PERMANOVA tests: plant litter *R*^2^ = 0.340, *p* = 0.001; soil *R*^2^ = 0.264, *p* = 0.001). PCO1 explained 49.8% and 34.8% of the total variation in plant litter and bulk soil, respectively, and clustered the samples by elevation.

In general, soil from high and low elevation differed in chemical composition affecting bulk soil communities. HE sites had high SOM content, which was correlated with high soil moisture content and NH_4_–N, TC, TN, and Na (Supplement S8) content. Soil bacterial composition was positively correlated when all soil parameters were included in the correlation (Mantel-*r* = 0.48, *p* = 0.001). Of all variables, the OM content and C/N ratio best described variance in soil bacterial community composition (Spearman, *r* = 0.68) and it was observed in the clustering pattern in Fig. 3d. Plant litter C:N was the only litter parameter measured, and it was higher at LE sites (lsmeans, *t*_(df = 4)_ = − 4.21, *p* = 0.014) (Supplement S9). However, this parameter was not correlated with variations in community composition in the plant litter bacterial communities (Mantel-*r* = − 0.018, *p* = 0.51).

### Host-Associated Microbiota and Its Link with Environmental Sources

**Fig. 4:**
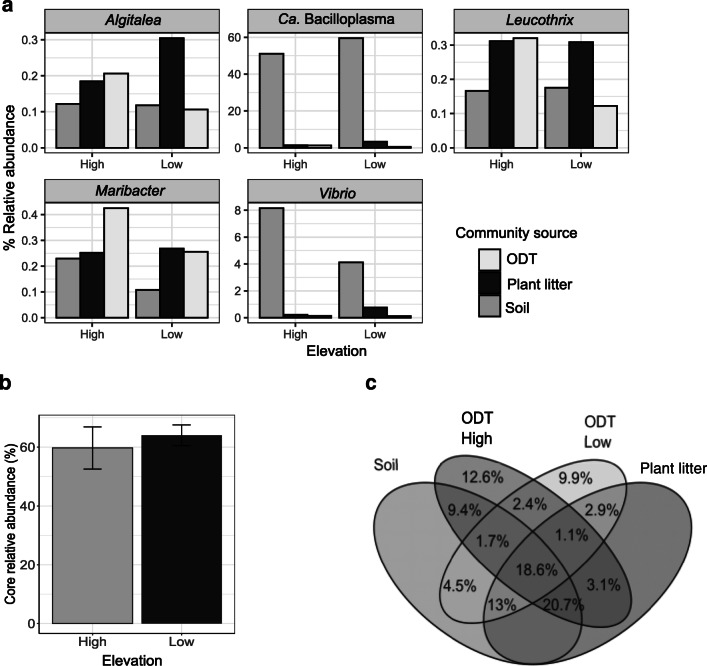
Interaction of bacterial communities in the *O. gammarellus* digestive tract (ODT) and the bacteria in environmental sources. **a** Relative abundances of the genera comprising the core bacteria and the sources in which they were observed at high and low elevations. **b** Total abundance of the core bacteria in the ODT bacterial community. **c** Venn diagram shows the percent of ASVs from the total ODT dataset that are exclusive to ODT or shared to external sources

The ODT core bacterial community, identified by their high prevalence (present in at least 90% of the ODT samples), was composed of 12 ASVs belonging to 5 genera: Ca. Bacilloplasma (7 ASVs), *Vibrio* (2 ASVs), *Leucothrix* (1 ASV), *Maribacter* (1 ASV), and *Algitalea* (1 ASV) (Fig. 4a). Core ODT bacteria were also found in soil and litter but in different abundances. *Ca. Bacilloplasma* and *Vibrio* were highly enriched in ODT compared to other sources (KW Dunn’s post hoc, Ca. Bacilloplasma: ODT-soil *Z* = 3.7, *p*_adj_ < 0.001, ODT-PL *Z* = 2.16, *p*_adj_ = 0.045; *Vibrio*: ODT-soil *Z* = 3.6, *p*_adj_ < 0.001, ODT-PL *Z* = 2.21, *p*_adj_ = 0.04) and were found more in plant litter than in soil. *Algitalea*, *Leucothrix*, and *Maribacter* were observed in low abundance in ODT, on average 0.12%, 0.17%, and 0.17%, respectively. Their abundance did not differ from that in soil and litter. Moreover, similar proportion of total core bacterial abundance was observed at both elevations (Wilcoxon test, *z* = 38, *p* = 0.86), representing on average 61.8% of their total abundance (Fig. 4b). Furthermore, from the total of the ASVs found in ODT samples, 18.6% (553 ASVs) were shared with both environmental sources, and both elevations represented 80.26% of the total ODT abundance. ASVs exclusively found in ODT accounted for 24.9% (669 ASVs) of the total and represented 1.41% of the total abundance (Fig. 4c). Part of this proportion was found at both elevations, but the majority was only found in either HE or LE. However, the two most representative families *Flavobacteriaceae* and *Rhodobacteriaceae* were found at both elevations (Fig. 4c, Supplement S10).

**Fig. 5:**
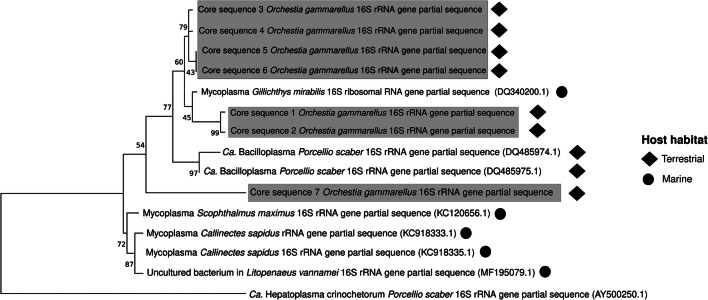
Phylogenetic relationships of Ca. Bacilloplasma found in digestive tracts. The maximum likelihood phylogenetic tree based on the partial 16S rRNA gene sequences indicates clusters related to host habitats. The numbers at the branches are confidence values based on bootstrap method (*B* = 1000 bootstrap iterations). The circles show the Ca. Bacilloplasma from hosts inhabiting terrestrial or semi-terrestrial habitats while diamonds hosts from marine habitats. The alphanumeric sequence at each node indicates the GenBank accession number. Gray highlight indicates sequences observed in present in 90% of the *O. gammarellus* digestive tract samples

Lastly, we explored the phylogenetic relationship of *Ca. Bacilloplasma* found in the ODT core (7 ASVs) with the most similar sequences obtained from the digestive tracts of other terrestrial crustaceans or marine animals (Fig. 5). All the bacterial taxa were very similar to each other; however, most of the *Bacilloplasma* associated with marine animals clustered together, while semi-terrestrial (*O. gammarellus*) and terrestrial (isopods) crustaceans formed another cluster.

## Discussion

### The Source Determines Bacterial Diversity and Composition

We found a rather low bacterial diversity in the digestive tract compared to the bulk soil and plant (shoot) litter. Similar low values of diversity were found in the hindgut of isopods [15] and in guts of several talidrid amphipods [8], suggesting that digestive tracts of terrestrial crustaceans form a rather unique environment. When compared to other talidrids, ODT was found to be less even with a predominance of *Mycoplasmataceae* and *Vibrionaceae* [8]. Moreover, ODT bacterial composition differed from that of both plant litter and bulk soil. On the one hand, the observed nestedness in ODT bacterial composition compared to the external environment indicates some sort of internal environmental filtering. On the other hand, the observed turnover when ODT is compared to soil and litter indicates that part of the ODT community is unique and adapted to the host internal environment. The absence of these ODT-associated bacteria in soil and litter suggests vertical transmission or the acquisition of these bacteria by cannibalism or coprophagy [42] and should be studied further.

Plant litter showed a lower bacterial diversity than soil, and we observed a high turnover between both sources. The low diversity is due to the lack of some phyla and enrichment of *Flavobacteriaceae*, *Chitinophagaceae*, and *Burkholderiaceae* in plant litter*.* The predominance of these three families in plant litter is related to their ability to degrade plant material or fungal mycelium under aerobic conditions [43, 44] implying an aerobic environment in plant litter. In contrast, aerobic and anaerobic conditions are found in bulk soil aggregates allowing also the presence of anaerobes [45]. For instance, the abundance of the anaerobic family Anaerolineae is higher in soils compared to plant litter.

### Elevation Drives the Soil and Plant Litter Bacterial Composition

Elevation, via seawater flooding frequency, determined the chemistry of soil and plant litter and their bacterial community. In this study, we observed that soil bacterial communities were driven by the SOM content and C/N ratio. These two parameters are linked in part to the vegetation zonation that occurs across salt marshes. Plant species with high tolerance to salinity are located at lower elevation and are gradually replaced by species with a lower tolerance across the elevational gradient [26]. This succession of plant species has an impact on soil properties, through the differential root exudation [46] and chemical composition of the litter, which is decomposed to soil organic matter [47]. This explains our observations of a higher C/N ratio in plant litter at LE compared to HE. Accordingly, we observed that similarity in dominant species resulted in comparable plant litter chemistry and soil properties. For instance, sites LB and LC shared the same dominant plant species (*E. atherica*, *A. maritima*, and *L. vulgare*) and were similar in the litter C/N ratio and microbial composition. Differences in vegetation composition possibly explain also the observed lower ASV richness in plant litter samples at LE.

### Digestive Tract Bacterial Community Composition

The most abundant genus in the core ODT was Ca. Bacilloplasma (family *Mycoplasmataceae*), which is highly adapted to gut environments, being attached to the gut cuticle in the terrestrial isopod *Porcellio scaber* [48]. Ca. Bacilloplasma is unculturable; therefore, studies that unravel its functional significance for the host have not been conclusive. Nevertheless, this bacterium seems not to be involved in lignocellulose digestion or in causing diseases, but rather in having a long-term commensal relationship with their host and a potential production of lactic and acetic acid [9, 48, 49]. Besides, its transmission has neither been fully described. Our study is the first to report this genus in plant litter and soil, although in much lower abundance than within the host, which is likely due to either excretion with the feces or remains of dead animals. Therefore, we suggest that this bacterium can be transmitted between amphipods through ingestion along with plant litter or cannibalism [50].

It is striking that *Bacilloplasma*-like bacteria have been found to successfully colonize guts of fish and terrestrial, semi-terrestrial, and marine crustaceans with different diets and that their dominance in each gut community is variable depending on the organism. Moreover, we observed that the phylogeny of these bacteria (based on the partial 16S rRNA gene) is linked to the environment of the host. Their presence in marine animals or in terrestrial animals with marine ancestry [50, 51] suggests a marine origin of this genus, and our results add information that likely host habitat is causing a species divergence. This divergence may be related to changes in diet and a close coevolution with the host, which is a subject for further studies.

The other core bacterial genera found in the digestive tract might be residents or acquired from the environment. The genus *Vibrio* had a high abundance and is likely a resident bacterium as it has been widely associated with marine crustaceans and marine environments [52]. Hence, given its semi-terrestrial nature, it is likely that the amphipod still preserves symbionts related to the marine environment. These symbionts probably facilitate the digestion of diatoms and microalgae, which are found in the amphipod gut [53]. Other members of the core bacterial community, although with lower abundance, were the genera *Maribacter*, *Leucothrix*, and *Algitalea*. These genera might be acquired from the environment because they are known for colonizing algae, soil, and rhizosphere [54–56]. Therefore, they are probably introduced along with the food the amphipod ingests. Thus, their high prevalence indicates that *O. gammarellus* prefers on feeding plant litter colonized by these bacterial genera and/or that they have a beneficial role in the degradation of marine and terrestrial vegetal material inside the host. Despite the efforts to minimize cross-contamination during the sample handling, it is important to note that some of the low abundant bacteria could have been incorporated during processing. Nevertheless, the members of the ODT bacterial core observed in this study represent genera that are mostly restricted to marine environments and not commonly found in negative controls [57].

Shared and unique taxa revealed the complexity of interactions between the bacterial communities associated with the host and the environment. The variable proportion correspondent to the non-core microbiome (~ 40% of total abundance) is distributed in ASVs shared between the three sources, in ASVs shared with only one source, or exclusively in ODT. Patterns of taxonomic composition are difficult to determine due to high variability between sites. We attribute this variability to site-specific characteristics—which determine soil and plant composition and, thus, the microbes that are interacting with the host—and to a high inter-individual variability in amphipods’ gut bacterial community [14, 58, 59]. Two taxa, *Flavobacteriaceae* and *Rhodobacteraceae*, contributed more to the abundance in the ODT exclusive bacterial community. Interestingly, these two genera have been used as biomarkers in different developmental stages in shrimp gut microbial communities [60], suggesting that some strains of these bacterial taxa are adapted to gut environments and that their presence might be linked to other host aspects that we did not specifically address in this study, such as specific age or sex.

Overall, our results suggest that local environmental conditions affect the bacterial communities in external sources, here plant litter and soil, which in part determine digestive tract composition of the host microbiome. However, the enrichment of specific potential symbionts is constant and in high abundance regardless of the local environmental conditions. The outcome of this stable proportion might determine the success in the performance and distribution of *O. gammarellus* at contrasting salt marsh elevations. Moreover, we propose *Bacilloplasmas* as overlooked co-evolutionary symbiont, which might provide pivotal functions for marine and semi-terrestrial animals.

## Supplementary Information

ESM 1 (DOCX 848 kb)

## Data Availability

All amplicon sequences are publicly accessible on the NCBI database under the BioProject ID PRJNA602740.
