# Supplementary material for: Linking Bacterial Communities Associated with the Environment and the Ecosystem Engineer Orchestia gammarellus at Contrasting Salt Marsh Elevations
Source: Microb Ecol. 2021 Jan 9;82(2):537–48. doi: 10.1007/s00248-020-01656-w (PMC8384807; doi:10.1007/s00248-020-01656-w)

**Appendix A**

**Linking bacterial communities associated with the environment and the ecosystem engineer Orchestia gammarellus at contrasting salt-marsh elevations**

Edisa García Hernández^1*^, Matty P. Berg^2,3^, A. Raoul van Oosten^2^, Christian Smit^3^, Joana Falcão Salles^1*^

^1^Microbial Community Ecology, Groningen Institute for Evolutionary Life Sciences, University of Groningen, Groningen, Netherlands

^2^Department of Ecological Sciences, Section Animal Ecology, Vrije Universiteit Amsterdam, Amsterdam, Netherlands

^3^Conservation and Community Ecology Group, Groningen Institute for Evolutionary Life Sciences, University of Groningen, Groningen, Netherlands

**A1. Soil and plant litter chemical parameters**

To analyze the differences in soil physicochemical parameters among elevations, we quantified soil moisture content, soil organic matter content (SOM), and the content of sodium (Na), total carbon (TC), total nitrogen (N), and nitrogen in nitrate (N-NO3-) and ammonium (N-NH4+). Soil moisture was measured by oven-drying 10 g of soil at 105°C for ~16 h. Moisture percentage was calculated as fresh weight minus dry weight, divided by fresh weight multiplied by 100. After that, the dried samples were placed in a muffle furnace (Naberthermn, Germany) at 550 °C for 4 h. The soil organic matter content was calculated as dry soil weight – dry weigh after ignition, divided by dry soil weight x 100 [1]. To measure N content in nitrate and ammonium, 12.5 g soil was mixed with 30 ml KCl (1M), shaken for ~16 h using a custom-made overhead shaker (1 turn/s). Afterwards, the suspension was filtered with a paper filter by gravity and the extract was analyzed for N-NO3- and N-NH4+ on a continuous flow auto analyzer ([2, 3], Type 5100; Skalar-40 BV, Breda, the Netherlands) using a colorimetric method [4]. For TC, TN and Na content, 10 g soil was first dried at 40 °C in a stove for 16 h and then ground to a fine powder in a Cyclotec 1093 mill. Sodium exchangeable ion content was measured by extraction of 5 g soil with ammonium acetate (1M, pH 7), mixed in the overhead shaker for 1 h and then filtered with a paper filter by gravity. The filtrate was analyzed on an atomic absorption spectrometer (AAS) (Varian Spectra AA 220FS, Australia). For TN and TC measurements, the soils were analyzed on a combustion elemental analyzer (CE Instruments EA 1110). For this measurement, one plot of the triplicates of HB was excluded because of low soil quantity and. Similarly, in site HC the analyzed samples were a composite sample of the three plots.

For TC and TN determination in plant litter samples, samples (2.5 g) from each plot were cleaned and dried at 70°C for 48 h, ground to a fine powder with a Retsch MM200 ball mill, after which 3-4 mg of material was analyzed in the elemental analyzer Flash EA112 (Thermo Scientific). To assess if plant litter %C:%N ratio differ among elevations we used t-test for data with equal variances using the package stats, prior normal distribution and homoscedasticity were tested.

**A2. Preparation of samples for sequencing**

To partially amplify the 16S rRNA gene, twenty-five µl PCR reactions were performed in triplicate using the FastStart High Fidelity (Roche) kit followed the protocol by [5] but using 10 ng of DNA sample. We used a 515F – 926R primer set, spanning variable region V4-5 as this region provide the best choice for phylogenetic assignments, richness estimates and longer fragments [6]. The forward primer also contained a barcode sequence (10-mer) to allow pooling of multiple samples in one sequencing run. Amplicon size was confirmed in 1% agarose gels, and the three PCR products of each sample were pooled together to reduce PCR bias and increase the coverage of 16S rRNA gene targets. Bulk soil PCR products were purified using the QIAquick PCR Purification Kit (Qiagen). For plant litter and digestive tract samples, two amplicon sizes (~400 and ~900 bp) were observed on the gel as these primers also can partially amplify 18S rRNA subunit [7]. Therefore, the purification was done using the QIAquick Gel Extraction kit (Qiagen) excising the expected DNA fragment (~400 bp). Amplicon fluorescence quantification was performed using the Quant-iT PicoGreen ds DNA assay kit (Invitrogen, Carlsbad, CA, USA) on a TECAN infinite M200 Pro (Maennedorf, Switzerland) plate reader using at 485 nm excitation and 535 nm emission. Amplicons from all samples were pooled in equimolar concentration (30 ng/sample) and sequenced at Genewiz (South Plainfield, USA) on an Illumina MiSeq sequencer using a 2 × 300-bp read configuration.

**A3. Total number of sequences obtained after removal of non-bacterial sequences and singletons.**

| **Source** | **Site** | **Sum of sequences** |
| --- | --- | --- |
| ODT | HA | 29711 |
|  | HB | 20528 |
|  | HC | 49882 |
|  | LA | 19616 |
|  | LB | 15527 |
|  | LC | 24411 |
| Plant litter | HA | 40099 |
|  | HB | 40879 |
|  | HC | 27788 |
|  | LA | 11952 |
|  | LB | 28926 |
|  | LC | 38211 |
| Soil | HA | 160915 |
|  | HB | 81212 |
|  | HC | 145961 |
|  | LA | 181097 |
|  | LB | 66142 |
|  | LC | 65831 |

**Literature cited**

1. Schulte B, Hopkins BG (1996) Estimation of soil organic matter by weight-loss-on-ignition. In: Magdoff FR (ed) Soil Organic Matter: Analysis and Interpretation. SSSA Spec. Publ., WI, USA, pp 349–359

2. Navone R (1964) Proposed method for nitrate in potable waters. J Am Water Works Assoc 56:781–783. https://doi.org/10.1002/j.1551-8833.1964.tb01270.x

3. Searle PL (1984) The Berthelot or Indophenol Reaction and Its Use in the Analytical Chemistry of Nitrogen A Review

4. Keeny DR, Nelson DW (1982) Nitrogen - inorganic forms. In: Page AL, Miller RH, Keeny DR (eds) Methods of soil analysis. ASA-SSSA, Madison, Wisconsin, USA, pp 643–698

5. Wang M, Yang P, Falcão Salles J (2016) Distribution of Root-Associated Bacterial Communities Along a Salt-Marsh Primary Succession. Front Plant Sci. https://doi.org/10.3389/fpls.2015.01188

6. Walters W, Hyde ER, Berg-Lyons D, et al (2016) Improved bacterial 16S rRNA gene (V4 and V4-5) and fungal internal transcribed spacer marker gene primers for microbial community surveys. mSystems 1:e00009-15. https://doi.org/10.1128/mSystems.00009-15

7. Parada AE, Needham DM, Fuhrman JA (2016) Every base matters: assessing small subunit rRNA primers for marine microbiomes with mock communities, time series and global field samples. Environ Microbiol 18:1403–1414. https://doi.org/10.1111/1462-2920.13023

**Supplementary materials**

**Linking bacterial communities associated with the environment and the ecosystem engineer Orchestia gammarellus at contrasting salt-marsh elevations**

Edisa García Hernández^1*^, Matty P. Berg^2,3^, A. Raoul van Oosten^2^, Christian Smit^3^, Joana Falcão Salles^1*^

^1^Microbial Community Ecology, Groningen Institute for Evolutionary Life Sciences, University of Groningen, Groningen, Netherlands

^2^Department of Ecological Sciences, Section Animal Ecology, Vrije Universiteit Amsterdam, Amsterdam, Netherlands

^3^Conservation and Community Ecology Group, Groningen Institute for Evolutionary Life Sciences, University of Groningen, Groningen, Netherlands

**Supplement S1**. Map showing the study area, the Dutch barrier Island of Schiermonnikoog (left panel) and the geographic location of the sampling sites on the old part of the salt marsh (black dots). Sites are coded as HA, HB, HC (high elevation, sites A-C) and LA, LB, LC (low elevation, sites A-C).

**
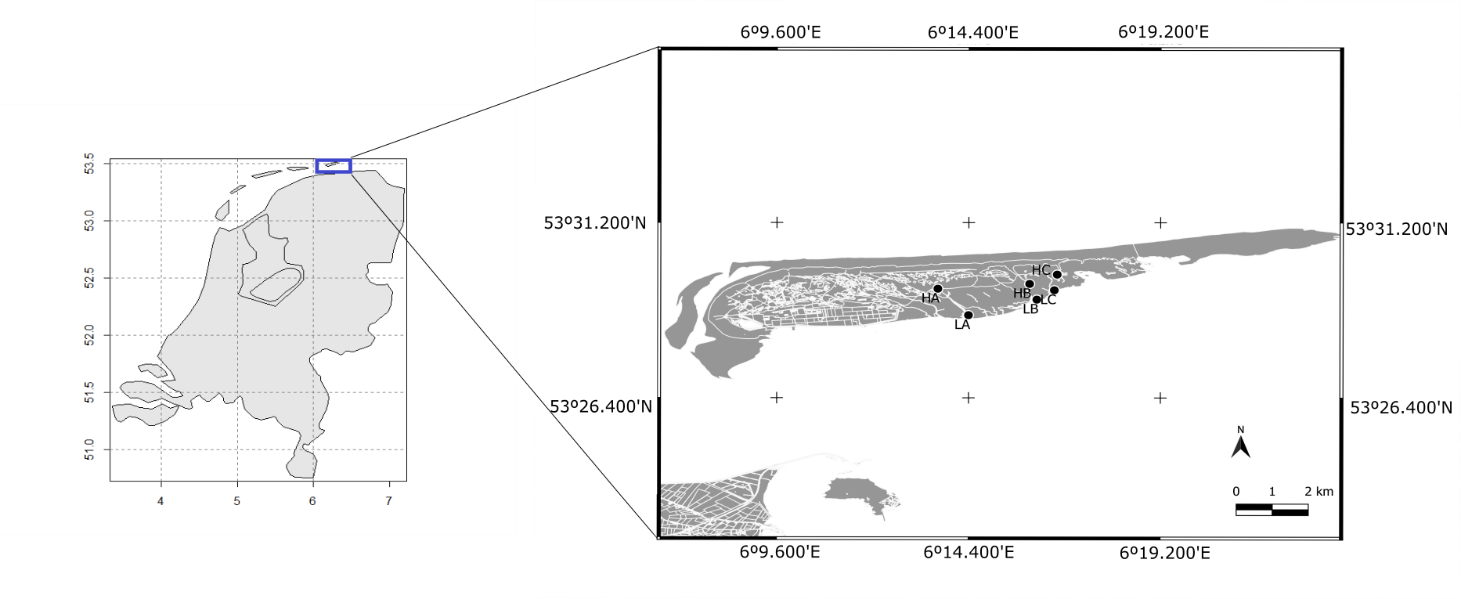
**

**Supplement S2.** Rarefaction curves of the observed bacterial ASVs (upper panel) and Shannon’s diversity index (lower panel) in the *Orchestia gammarellus* digestive tract (ODT, in pink), in plant litter (green) and soil (blue).


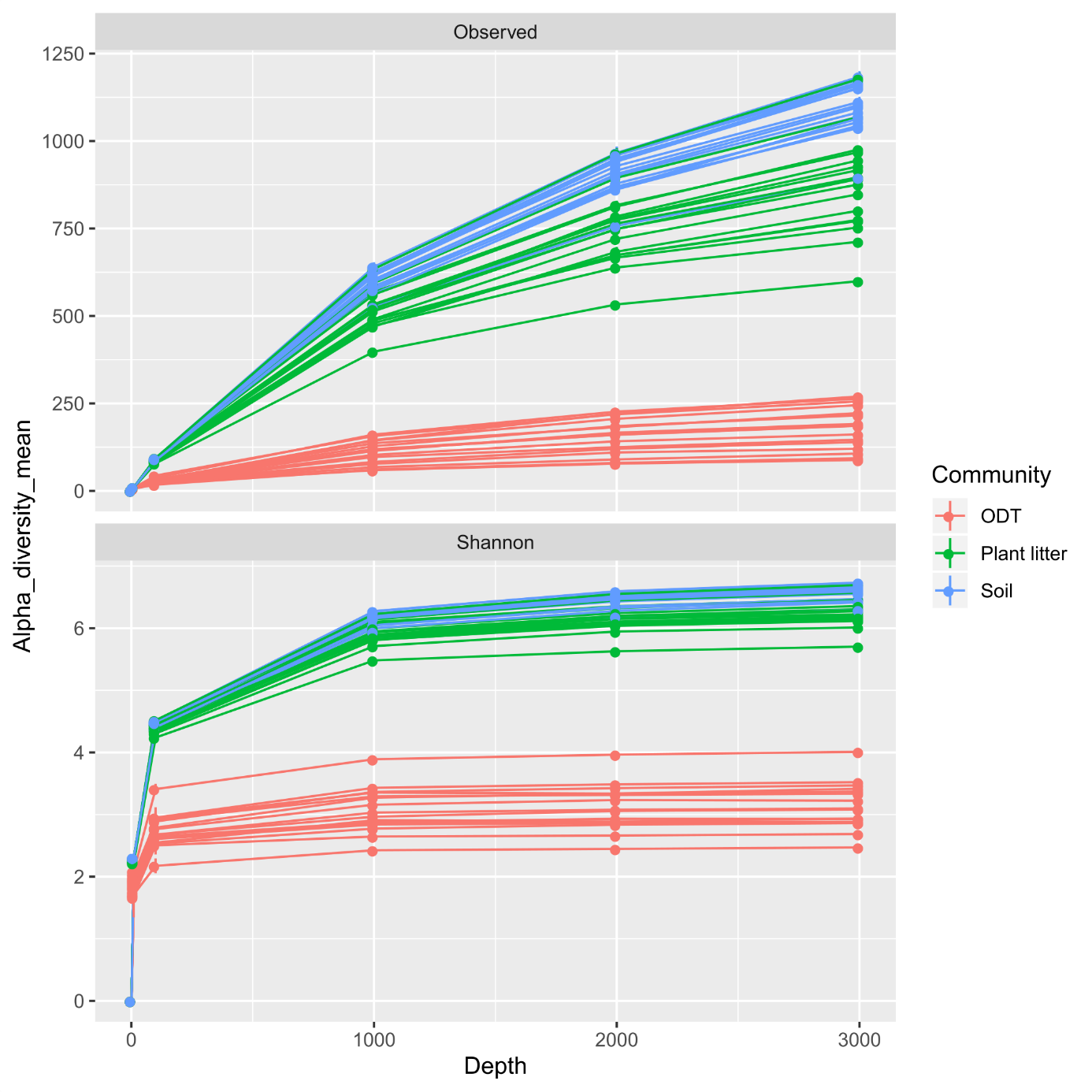


**Supplement S3**. Summary statistics of the comparison (group 1 versus group 2) of bacteria ASV richness, ASV Shannon diversity and ASV phylogenetic distance between soil, plant litter and/or *Orchestia gammarellus* digestive tract (ODT). All parameters were calculated based on ASVs obtained from a rarefied dataset to the 3000 sequences depth. The mean of the replicates was obtained and community effect was tested with ANOVA and Post-hoc comparison of least-square means.

| **Richness (Observed ASVs)*** | | | | | | |
| --- | --- | --- | --- | --- | --- | --- |
| Group 1 | Group2 | estimate | SE | df | t.ratio | *p* value adj Tukey |
| ODT | Plant litter | -905 | 79.6 | 15 | -11.4 | <0.001 |
| ODT | Soil | -1283 | 79.6 | 15 | -16.1 | <0.001 |
| Plant litter | Soil | -387 | 79.6 | 15 | -4.6 | 0.0009 |
| * Normality test: Shapiro test W=0.96, *p*=0.6129 | | | | | | |
|  | | | | | | |
| **Shannon Diversity Index**** | | | | | | |
| Group 1 | Group 2 | estimate | SE | df | t.ratio | *p* value adj. Tukey |
| ODT | Plant litter | -3.31 | 0.119 | 15 | -27.8 | <0.001 |
| ODT | Soil | -3.72 | 0.119 | 15 | -31.7 | <0.001 |
| Plant litter | Soil | -0.402 | 0.119 | 15 | -3.4 | 0.0109 |
| ** Normality test: Shapiro test W=0.94, *p*=0.35 | | | | | | |
|  | | | | | | |
| **Faith Phylogenetic Distance***** | | | | | | |
| Group 1 | Group 2 | estimate | SE | df | t.ratio | *p* value adj. Tukey |
| ODT | Plant litter | -34.8 | 2.75 | 15 | -12.7 | <0.001 |
| ODT | Soil | -53.6 | 2.75 | 15 | -19.5 | <0.001 |
| Plant litter | Soil | -18.8 | 2.75 | 15 | -6.8 | <0.001 |
| *** Normality test: Shapiro test W=0.99, *p*=0.99 | | | | | | |

**Supplement S4.** PCoA curves of bacterial communities in bulk soil, litter and digestive tract of *Orchestia gammarellus*. Source type, i.e. soil, litter and digestive tract, structures bacterial community composition, based on taxonomy (upper panel) or phylogeny (lower panel). PCoA of Bray Curtis and UniFrac weighted (A and B, respectively) distances for *Orchestia gammarellus* digestive tract (ODT), plant litter and soil samples. Symbols represent elevation and are colored by source type. The first two PCOs are plotted with the percentage of variation explained by each PCO. Ellipses indicate similarity at 95% confidence cluster elevations.

**A.**


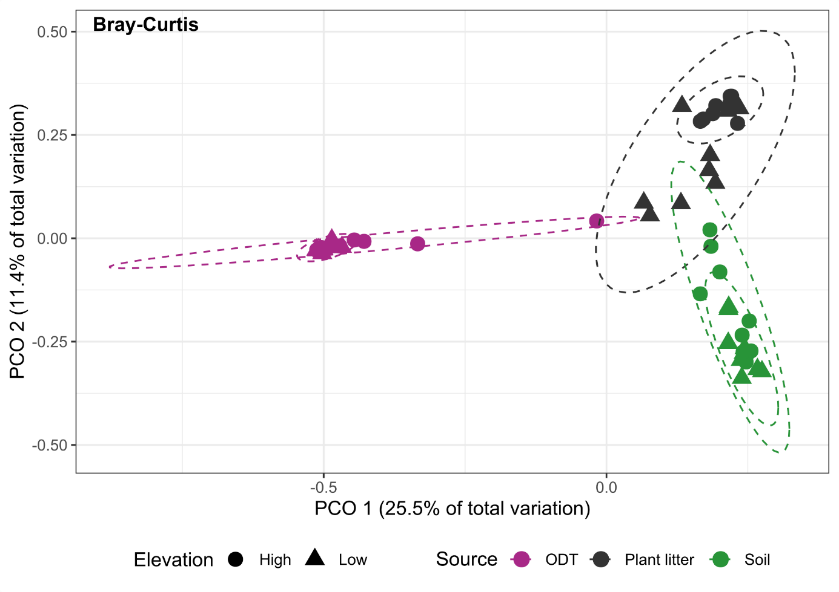


**
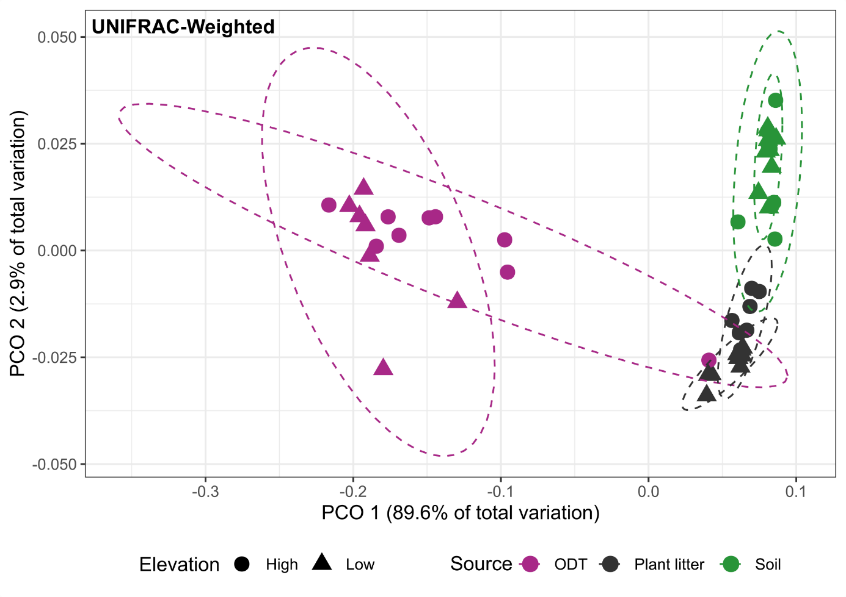
 B.**

**Supplement S5.** Summary of permutational multivariate analysis of variance (PERMANOVA, 999 permutations) testing the dissimilarity difference between soil, litter and digestive tract bacterial composition.

| **Distance matrix** | **Pseudo-F** | **Df** | **R^2^** | **Significance**  **(*p* value)** | **Significance dispersion of data (*p* value)** |
| --- | --- | --- | --- | --- | --- |
| Bray-Curtis | 12.69 | 2 | 0.346 | 0.001 | 0.001 |
| Unweighted  UniFrac | 2.05 | 2 | 0.215 | 0.001 | 0.603 |
| Weighted  UniFrac | 117.30 | 2 | 0.830 | 0.001 | 0.021 |

**Supplement S6.** Results of applying the Dirichlet multinomial mixture-based model in the total ASV dataset. In the upper panel is represented the fitting optimal classification in three types of bacterial communities (envirotypes). In the lower table are indicated the number of samples of soil, litter and *O. gammarellus* digestive tract (ODT) corresponding to each envirotype and their most predominant bacterial families.


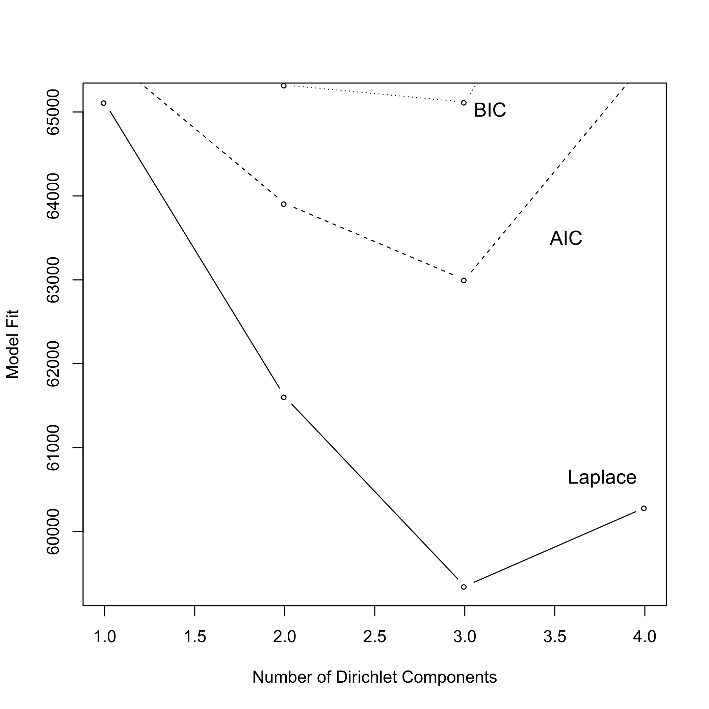


| **Envirotype** | **Predominant families** | **ODT** | **Plant litter** | **Soil** |
| --- | --- | --- | --- | --- |
| 1 | *Flavobacteriaceae/Chitinophagaceae* | 1 | 18 | 0 |
| 2 | *Flavobacteriaceae/Anaerolinaceae* | 0 | 0 | 18 |
| 3 | *Mycoplasmataceae/Vibrionaceae* | 17 | 0 | 0 |

**Supplement S7**. Summary statistics of the comparison of the alpha diversity based on ASVs across elevations. HE = high elevation sites, LE = low elevation sites, ODT = *O. gammarellus* digestive tract. The factor “site” was considered as random factor in the linear mixed model. The degrees of freedom method applied was Kenward-roger. Significant values of the fdr *p* adjustment method for 2 test (*p* value < 0.05) are indicated in bold.

| **Richness** | | | | | | | | |
| --- | --- | --- | --- | --- | --- | --- | --- | --- |
| Source | Group | Group | estimate | | SE | df | t.ratio | p.value |
| ODT | HE | LE | -26.1 | | 56.8 | 3.8 | -0.46 | 0.67 |
| Plant litter | HE | LE | 317 | | 98.2 | 3.94 | 3.23 | **0.03** |
| Soil | HE | LE | -6.78 | | 80.9 | 4 | -0.08 | 0.94 |
|  |  |  |  |  |  |  |  |  |
| **Shannon's Diversity index** | | | | | | | | |
| Source | Group | Group | estimate | | SE | df | t.ratio | p.value |
| ODT | HE | LE | -0.06 | | 0.24 | 3.68 | -0.26 | 0.81 |
| Plant litter | HE | LE | 0.39 | | 0.11 | 3.96 | 3.64 | **0.02** |
| Soil | HE | LE | -0.05 | | 0.1 | 4 | -0.50 | 0.64 |
|  |  |  |  |  |  |  |  |  |
| **Faith's Phylogenetic index** | | | | | | | | |
| Source | Group | Group | estimate | | SE | df | t.ratio | p.value |
| ODT | HE | LE | -0.29 | | 2.78 | 3.55 | -0.10 | 0.92 |
| Plant litter | HE | LE | 9.53 | | 3.65 | 3.9 | 2.61 | 0.06 |
| Soil | HE | LE | 4.85 | | 3.57 | 4 | 1.36 | 0.25 |

**Supplementary material S8.** Soil physicochemical parameters across sampling sites. Sites are coded as HA, HB, HC (high elevation, sites A-C) and LA, LB, LC (low elevation, sites A-C). Average values and standard error are given for soil moisture (%), Soil organic matter content (%), and NO_2_/NO_3_-N, NH_4_-N, and Na content (mg kg^-1^) and C content. For more details of the sites, see Table 1.

| Site | Moisture (%) | Organic matter content (%) | Nitrates  (mg N kg-1 soil) | Ammonium  (mg N kg^-1^ soil) | C /N ratio | Na (mg/ kg^-1^ dry soil) |
| --- | --- | --- | --- | --- | --- | --- |
| HA | 49.0 ± 0.9 | 17.7 ± 0.3 | 46.1 ± 4.5 | 1.0 ± 0.4 | 11.1 ± 0.2 | 727.8 ± 5.0 |
| HB | 69.8 ± 3.5 | 29.5 ± 4.6 | 155.9 ± 35.1 | 30.6 ± 10.4 | 11.6 ± 0.8 | 1354.0 ± 34.4 |
| HC | 73.2 ± 0.5 | 35.0 ± 2.0 | 111.3 ± 19.0 | 35.7 ± 8.8 | 12.8 | 1544.6 |
| LA | 50.3 ± 0.7 | 16.9 ± 1.0 | 37.9 ± 1.3 | 1.3 ± 0.5 | 10.8 ± 0.5 | 665.4 ± 29.6 |
| LB | 49.7 ± 0.2 | 14.6 ± 0.4 | 134.8 ± 18.6 | 8.8 ± 2.6 | 13.0 ± 0.1 | 641.1 ± 14.8 |
| LC | 52.6 ± 0.2 | 14.1 ± 0.1 | 112.3 ± 15.6 | 14.3 ± 6.1 | 13.4 ± 0.1 | 791.6 ± 10.2 |

**Supplement S9**. Differences in the carbon/nitrogen content ratio in plant litter samples from high and low salt marsh. Barplots indicate the average of three sampling plots and different letters indicate significant differences among elevations (lsmeans, *t* = -4.21, df = 4, *p*=0.014).


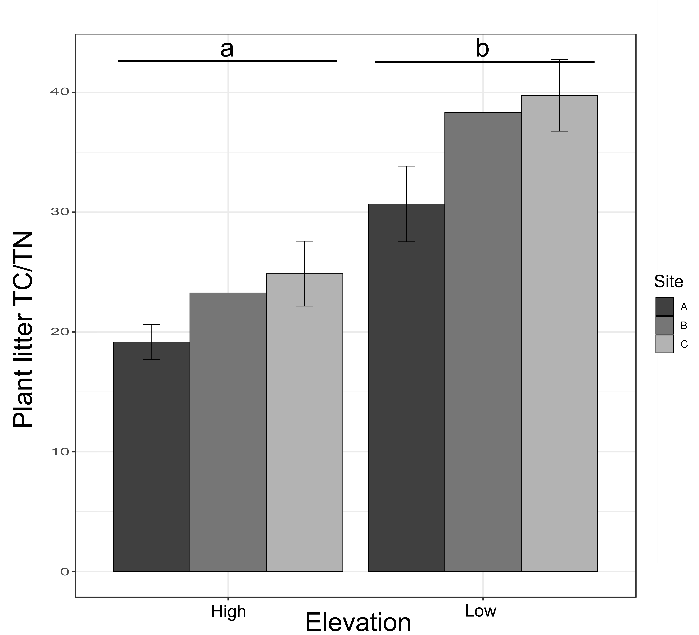


**Supplement S10.** Abundance of the bacteria taxa exclusively found in ODT samples and not in environmental sources at high and low elevations.


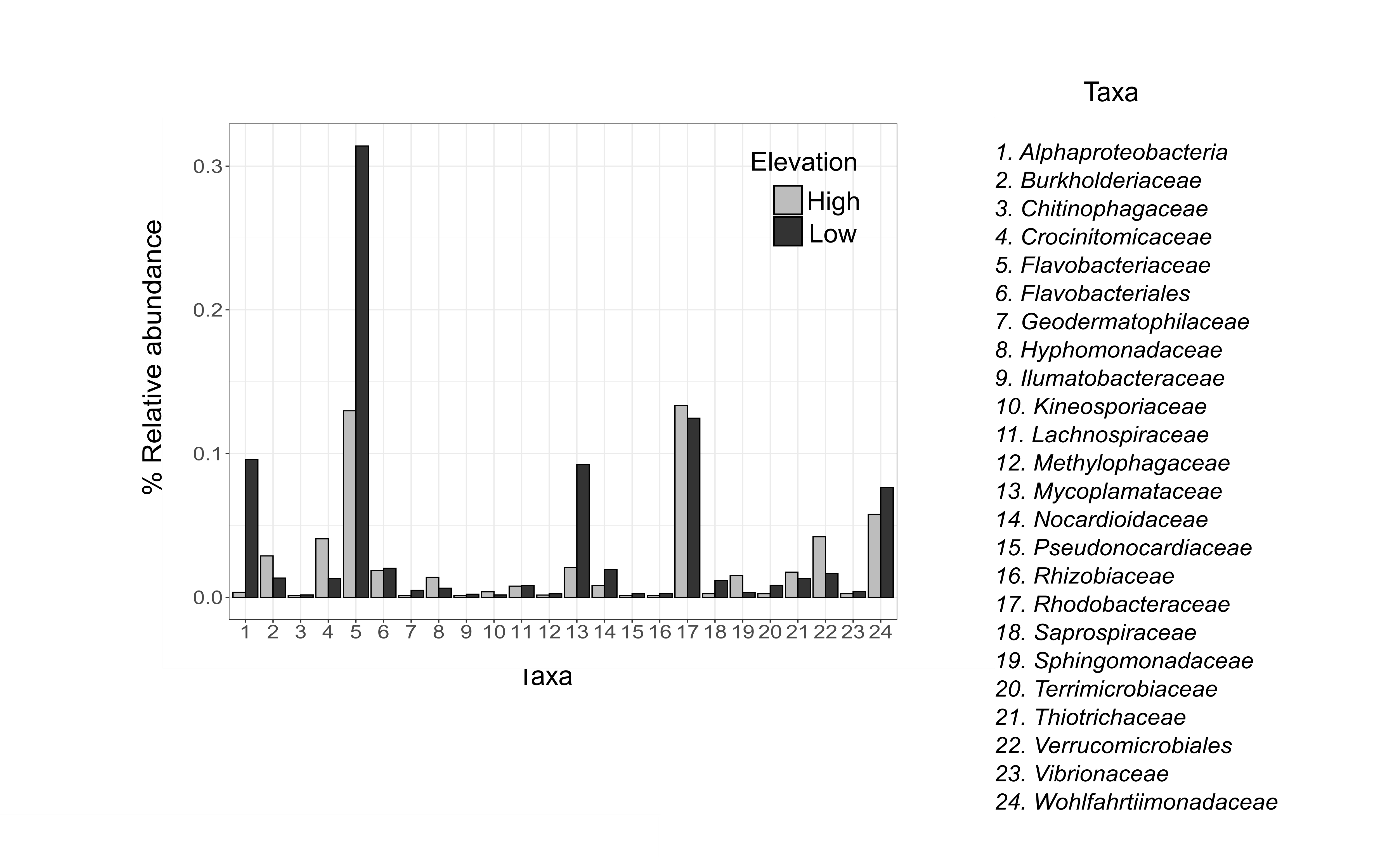

Supplement: Supplementary file 1 — (DOCX 848 kb) [file 248_2020_1656_MOESM1_ESM.docx]
